# Supplementary material for: Profiling Virus-Specific Tcf1+ T Cell Repertoires During Acute and Chronic Viral Infection
Source: Front Immunol. 2020 May 29;11:986. doi: 10.3389/fimmu.2020.00986 (PMC7272574; doi:10.3389/fimmu.2020.00986)
Supplement: Supplementary file 1 [file Data_Sheet_1.PDF]

Supplementary material for “Profiling virus-specific Tcf1+ T cell repertoires during acute and chronic viral infection”, Yermanos et al.

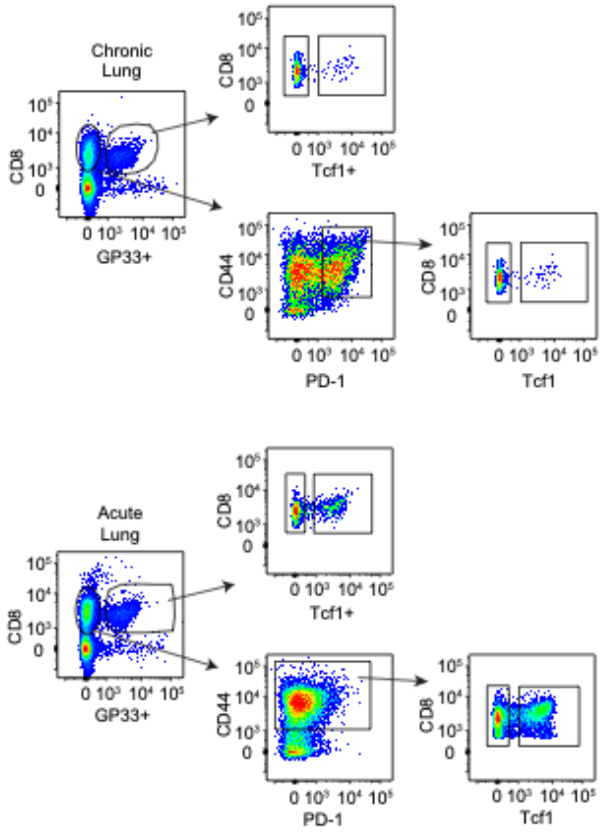

Figure S1. Example sorting strategy and representative FACS plots for lungs of chronically and acutely LCMV infected *Tcf7*^{GFP} mice.

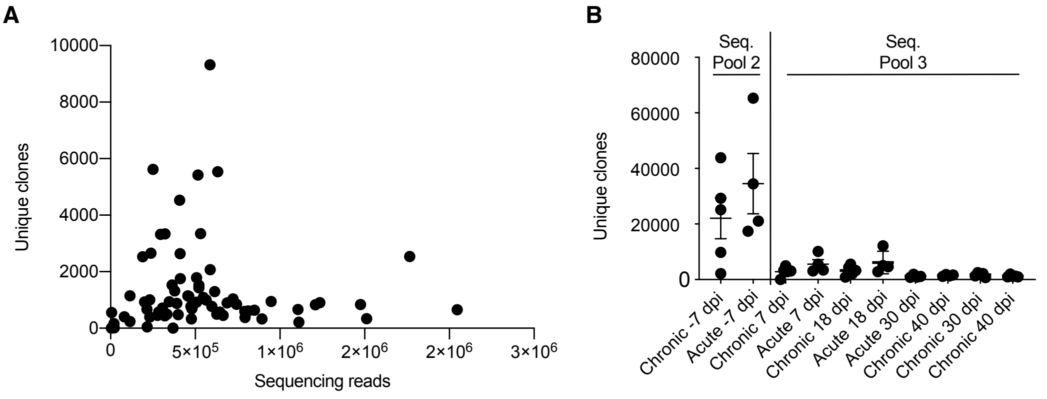

Figure S2. No correlation between sequencing depth and number of unique clones. (A) The number of unique clones (defined by unique CDR3b amino acid sequence) plotted against the number of recovered sequencing reads for each spleen/LN and lung repertoire 40 days post infection (dpi) following either acute or chronic LCMV infection. (B) The number of unique clones from the unsorted blood repertoires following acute or chronic LCMV infection.

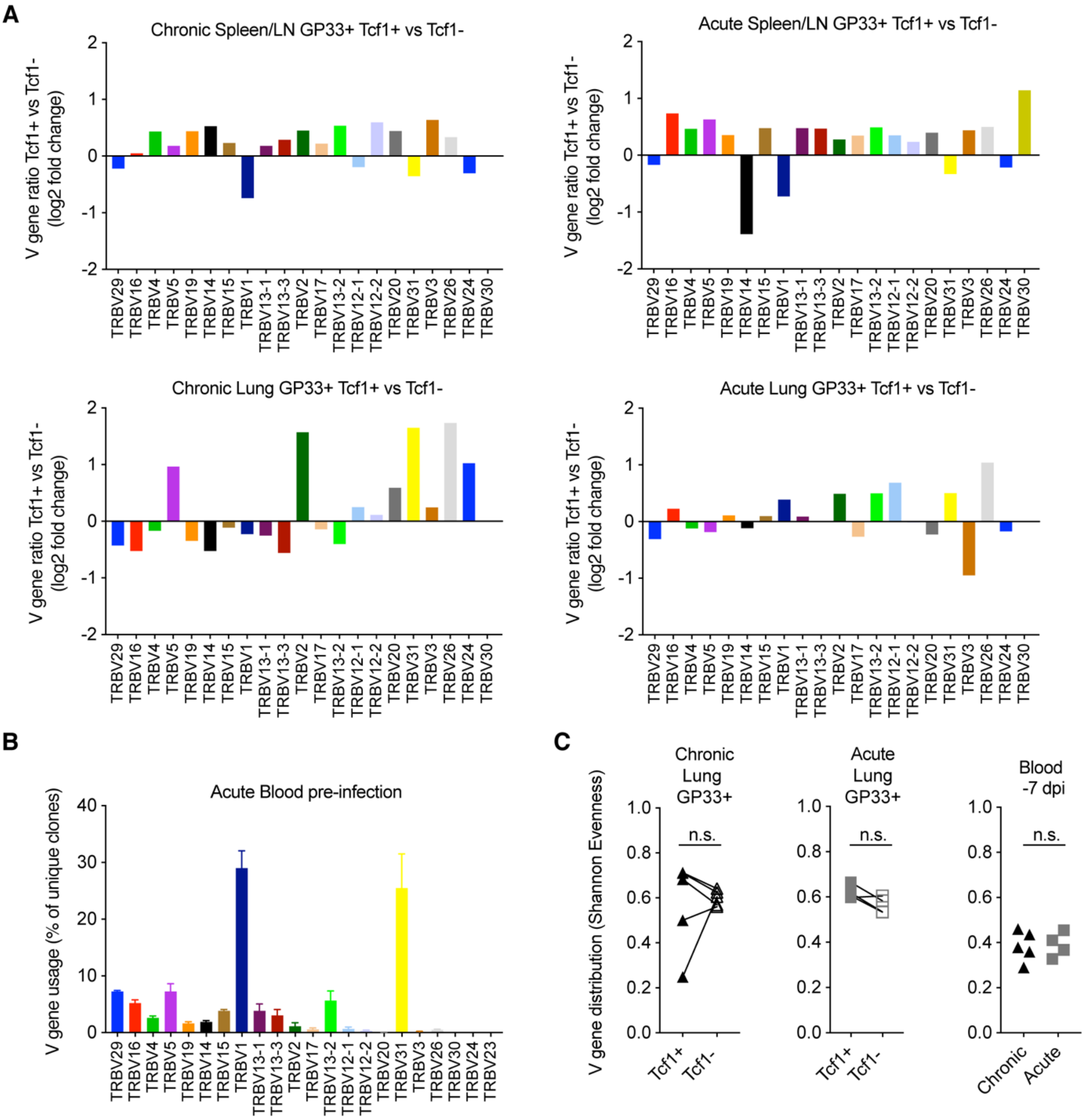

Figure S3. Minor changes in V gene usage between Tcf1+ and Tcf1- repertoires. (A) The log_2 change between the Tcf1+ and Tcf1- repertoires for each V gene from sorted spleen/LN and lungs 40 dpi. (B) V gene usage (percent of unique clones) for the acute blood repertoire before any viral infection. (C) Shannon evenness for V gene usage for GP33+ lung repertoires 40 dpi and blood repertoires 7 days before viral infection. * p≤0.05, ** p≤0.05, *** p≤0.005, **** p≤0.0005, not significant (n.s.) p>0.05.

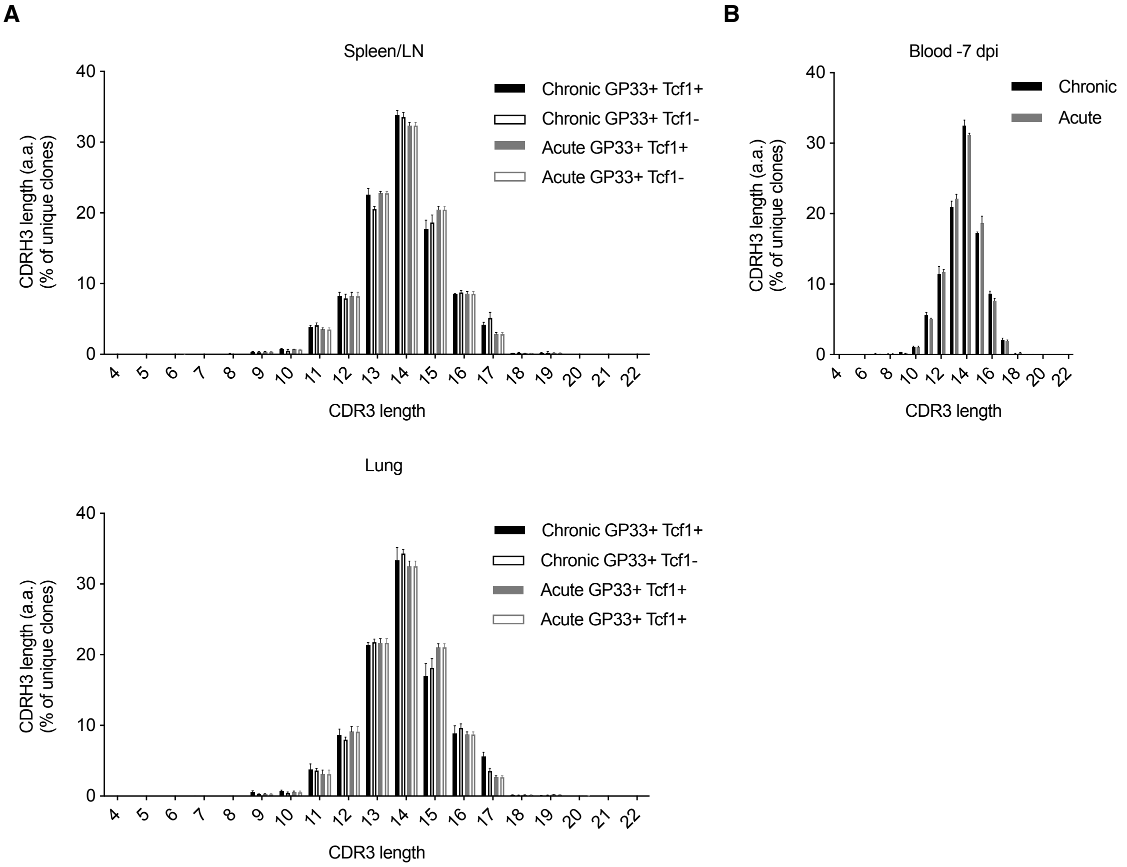

Figure S4. Similar CDR3b length distribution across Tcf1+ and Tcf1- repertoires. (A) CDR3b length distribution for (A) Spleen/LN and lung 40 days post infection (dpi) and (B) blood before any viral infection.
